# Diagnosis and treatment of anaphylaxis: there is an urgent needs to implement the use of guidelines

**DOI:** 10.1590/S1679-45082017RW4089

**Published:** 2017

**Authors:** Maria Luiza Kraft Köhler Ribeiro, Herberto José Chong, Nelson Augusto Rosario

**Affiliations:** 1 Universidade Federal do Paraná, Curitiba, PR, Brazil

**Keywords:** Anaphylaxis/diagnosis, Anaphylaxis/drug therapy, Epinephrine/therapeutic use, Anafilaxia/diagnóstico, Anafilaxia/tratamento farmacológico, Epinefrina/uso terapêutico

## Abstract

Anaphylaxis is a severe, life-threatening generalized or systemic hypersensitivity reaction that requires rapid and adequate care. This study aimed to obtain an integrated view of the level of physicians' knowledge related with treatment of anaphylaxis in studies published within the last 5 years. Sixteen studies were found and four points were identified as of the great interest to the authors: (1) emergency pharmacological treatment, (2) epinephrine auto-injectors prescription, (3) knowledge of the main signs of anaphylaxis, and (4) admission of the patient to verify biphasic reactions. Concern about the use of intramuscular adrenaline as the first choice in relation with anaphylaxis was evident in most studies, rather than its use in the comparison dial, and especially low in a study that included data from Brazil, in which the frequency of its use was 23.8%. An adrenaline autoinjector is highly recommended among specialists for patients at risk of anaphylaxis, however, its use is still infrequent among non-specialists and in countries that this agent is not available. Intervention studies have shown improved medical knowledge of anaphylaxis following disclosure of the information contained in the international guidelines. The analysis of these studies reinforces the need to disseminate international guidelines for diagnosis and treatment of anaphylaxis, as well as providing an adrenaline autoinjector, to improve management and to prevent a fatal outcome.

## RESUMO

Anafilaxia é uma reação de hipersensibilidade generalizada ou sistêmica grave, com risco de morte, que exige atendimento rápido e correto. Este estudo teve como objetivo obter uma visão integrada do nível de conhecimento dos médicos no atendimento da anafilaxia à luz dos estudos publicados internacionalmente nos últimos 5 anos. Foram encontrados 16 estudos, com quatro pontos identificados como de maior interesse dos autores: (1) tratamento farmacológico de emergência, (2) prescrição de autoinjetores de adrenalina, (3) conhecimento dos principais indícios da anafilaxia e (4) observação do paciente para verificar reações bifásicas. A preocupação com o uso da adrenalina intramuscular como primeira escolha frente à anafilaxia foi evidente na maioria dos estudos, mas o conhecimento sobre seu uso se mostrou desigual e especialmente baixo em estudo que incluiu dados do Brasil, onde a frequência de seu uso foi de 23,8%. A adrenalina autoinjetável é altamente recomendada entre especialistas para pacientes em risco de anafilaxia, mas seu uso ainda é pouco frequente entre não especialistas e em países que não dispõem dela em seus mercados internos. Estudos de intervenção comprovaram a melhora no entendimento dos médicos sobre anafilaxia após a divulgação das informações contidas nas diretrizes internacionais. A análise dos estudos reforça a necessidade de disseminar as diretrizes internacionais no manejo da anafilaxia, bem como de disponibilizar a adrenalina autoinjetável, a fim de melhorar o atendimento e evitar um desfecho fatal.

## INTRODUCTION

Anaphylaxis constitutes a generalized or severe systemic hypersensitivity reaction with risk of death.^(^ ^1^ ^)^ This is the most life-threatening emergency clinical conditions both by unpredictability of its appearance and potential severity of its progression.^(^ ^2^ ^)^

The Immunoglobulin E (IgE) mediated anaphylaxis can be triggered by a number of environmental factors, such as medications, food, insect poison, latex and physical stimulation.^(^ ^3^ ^)^ The incidence and prevalence of anaphylaxis have increased in the last decade.^(^ ^4^ ^)^

Anaphylaxis affects at least two systems, including skin and mucosae (80 to 90% of cases) and respiratory system (70% of cases), in addition to gastrointestinal tract and cardiovascular system in which immediate administration of intramuscular (IM) adrenaline is need as first-line therapy for symptoms reversion.^(^ ^3^ ^)^

In addition to reverse the clinical emergency picture, there is the need to prevent episodes, and provide guidance for patients and their families concerning actions to prevent fatal outcome.

The behavior of professionals facing anaphylaxis is a crucial point. Physicians are expected to delivery rapid and adequate care. The World Allergy Organization (WAO) has developed guidelines for assessment and management of anaphylaxis in order to standardize care.^(^ ^1^ ^)^

Although a number of guidelines have been organized by specialty societies for diagnosis and management of anaphylaxis, studies have shown that physicians' knowledge about this affection diverges in the different regions analyzed in our study.

## OBJECTIVE

To determine physicians' knowledge regarding anaphylaxis care according to aspects searched by international authors.

## METHODS

This was an integrative review that assessed the knowledge of physician regarding anaphylaxis. Data were searched in December 2016 in PubMed, which includes the Medical Literature Analysis and Retrievel System Online (Medline), also in the Virtual Health Library (VHL), Scientific Electronic Library Online (SciELO), and Latin-American and Caribbean System on Health Sciences Information (LILACS).

We included studies published between 2012 and 2016. Keywords used in Portuguese and English from DeCS/MeSH terms were “anaphylaxis” AND “therapy” AND “knowledge”.

We did not include in our sample case reports, studies not related with assess of knowledge on anaphylaxis or studies including professionals different from the medical area, practical guidelines for clinical management were also excluded.

After reading the abstracts, a database was created including the studies that approached aspects regarding knowledge of physician on anaphylaxis. The next steps involved the reading of articles full text, their organization into main topics approached by authors and comparison with most relevant results that we have found.

## RESULTS

Our search retrieved 105 articles from PubMed, 11 from VHL, 3 from SciELO and 2 from LILACS. After applying the exclusion criteria, we selected 16 studies from the PubMed, 1 from VHL, and no studies from SciELO and LILACS. The two publications previously found in LILACS were guidelines containing direct emergency actions. In SciELO two were practical guidelines and one was a case report. Still, the single study that approached the knowledge of physician on anaphylaxis found in VHL was a duplicate publication from PubMed.

The majority of publications used quantitative approach (15 studies) and only one used the qualitative approach. Most of the studies were published in the United States (7), followed by Turkey (4) ( Table 1 ).

**Table 1:** Assessment of knowledge of physicians and health professional regarding anaphylaxis between 2012 and 2016

Authors	Sample composition	Local	N
Droste et al.,^(^ ^5^ ^)^	Hospitalists	England	284
Jacobsen et al.,^(^ ^6^ ^)^	Paramedics	The United States	3,537
Kahveci et al.,^(^ ^7^ ^)^	Residents in Pediatrics and Family Medicine	Turkey	38
Fineman et al.,^(^ ^8^ ^)^	Allergists	The United States	500
Solé et al.,^(^ ^9^ ^)^	Allergists and Immunologists, and non-specialized physicians	23 Ibero-american countries	510
Desjardins et al.,^(^ ^10^ ^)^	Allergists and non-specialized physicians	Canada	727
Erkoçoğlu et al.,^(^ ^11^ ^)^	Primary care physicians	Turkey	297
Baççioğlu et al.,^(^ ^12^ ^)^	Non-allergists, internists, medical students, nurses and paramedics	Turkey	1,172
Grossman et al.,^(^ ^13^ ^)^	Pediatricians from Pediatrics	The United	620
	Emergency Service	States	
Manivannan et al.,^(^ ^14^ ^)^	Analysis of Electronic Records	The United States	202
Wang et al.,^(^ ^15^ ^)^	Electronic questionnaire (physicians)	The United States and other 142 countries	2,882
Derinoz et al.,^(^ ^16^ ^)^	Pediatricians participating in two congresses	Turkey	410
Fineman et al.,^(^ ^17^ ^)^	Qualitative	The United States	-
Altman et al.,^(^ ^18^ ^)^	Allergists, immunologists, emergency pediatric and family medicine physicians	The United States	316
Manuyakorn et al.,^(^ ^19^ ^)^	Analysis of medical records	Thailand	160
Plumb et al.,^(^ ^20^ ^)^	Early-career physicians	The United Kingdom	78 (2002); 68 (2013)

Qualitative finding created by specialists of the American Academy of Allergy, Asthma & Immunology (AAAAI), discussed the current knowledge regarding anaphylaxis and they have highlighted three negative points in care in this clinical picture: (1) complexity of diagnosis, (2) prescription of adrenaline auto-injector (3) inadequate follow-up.^(^ ^17^ ^)^ The content analysis of quantitative studies pointed out the main questions raised by authors, such as: (1) pharmacological treatment of anaphylaxis emergency, (2) prescription of adrenaline auto-injector, (3) knowledge of main signs and symptoms and (4) observation of patient after resolution of the anaphylactic picture.

### Adrenaline as the first treatment option

Of the 16 studies included, 12 approached the frequency of adrenaline (epinephrine) use by physicians, and 4 reported results focused on specialty of allergy and immunology.

Option for adrenaline as the first treatment choice for anaphylaxis was mentioned by frequency that varied from 81 to 98% among physician in the United States of different specialties,^(^ ^18^ ^)^ and 93% among allergists/immunologists. The number of allergists/immunologists from the United States, who used the adrenaline as first treatment option (97%) also agreed with another study.^(^ ^8^ ^)^ Other two studies reported data related to allergists and non-allergists specific concerning IM adrenaline. In a study involving 23 ibero-american countries, including Brazil, the use of IM adrenaline was mentioned by 71.11% of specialists in allergy/immunology.^(^ ^9^ ^)^ Still, a study carried out in Canada verified that allergists are almost four times more likely to have this pharmacological behavior than non-allergists (odd ratio – OR=3.8; 95% confidence interval 95%CI: 1.43-10.11).^(^ ^10^ ^)^

A Canadian study reported that old physician were slightly less likely to recommend the use of IM adrenaline (OR=0.98; 95%CI: 0.97-0.99), and this was the unique study to be associated with professional age and use of intramuscular adrenaline.^(^ ^10^ ^)^ In eight studies, we observed data from non-allergists and in only one there were no specifications concerning IM adrenaline in the guiding question. An intervention study carried out in a hospital in the United States showed that only 33% of physician from the emergency service prescribed adrenalin as the first-line care – frequency reached was 51% after implantation of a guideline in the studied service.^(^ ^14^ ^)^

The remaining studies had a broadly view of results. The use of IM adrenaline was more frequent in Thailand in which an analysis of medical records reported that 93.8% of children with anaphylaxis were treated with medications and by this route,^(^ ^19^ ^)^ this result was followed by England in a study in two hospitals with 79.5% and 75.6% that study frequency of physicians, respectively.^(^ ^5^ ^)^

In the United States, 66.9% of participants elected IM adrenaline,^(^ ^13^ ^)^ placed between two analyzed samples in the United Kingdom from 45% in 2002, and 74% in 2013.^(^ ^20^ ^)^ The frequency found by a study carried out in Turkey was 43.3% among primary care physicians,^(^ ^11^ ^)^ followed by American paramedics with 38.9%.^(^ ^6^ ^)^

Low frequencies than 30% of IM adrenaline use were checked in two samples: the first was obtained in Turkey composed by non-allergists, internists, medical students, nurses, and paramedics with 29%,^(^ ^12^ ^)^ and the second study already mentioned that involved ibero-american countries and found that 23.8% of non-specialized physician that mentioned to elect IM adrenaline as the first-line option for anaphylaxis^(^ ^9^ ^)^ ( Table 2 ).

**Table 2:** Adrenaline as the first choice for anaphylaxis treatment among non-specialist and specialist physicians

Authors		Frequency of use	Administration route	Country
Specialists in allergy and immunology				
	Fineman et al.,^(^ ^8^ ^)^	97.0%	Not specified	The United States
	Solé et al.,^(^ ^9^ ^)^	71.1%	Intramuscular	ibero-American countries
	Desjardins et al.,^(^ ^10^ ^)^	Allergists prescribed adrenaline 3.8 times more than non-allergists (OR=3.8; 95%CI: 1.43-10.11)	Intramuscular	Canada
	Altman et al.,^(^ ^18^ ^)^	Between 93.0% and 98.0% (pediatrician and internal medicine, respectively)	Not specified	The United States
Non-specialists in allergy and immunology				
	Droste et al.,^(^ ^5^ ^)^	79.5% and 75.6% (hospitals A and B, respectively)	Intramuscular	England
	Jacobsen et al.,^(^ ^6^ ^)^	Paramedics 38.9%	Intramuscular	The United States
	Solé et al.,^(^ ^9^ ^)^	23.8%	Intramuscular	PIbero-American Countries
	Erkoçoğlu et al.,^(^ ^11^ ^)^	43.3%	Intramuscular	Turkey
	Baççioglu et al.,^(^ ^12^ ^)^	Non-allergists, internists, medical students, nurses and paramedics 29%	Intramuscular	Turkey
	Grossman et al.,^(^ ^13^ ^)^	66.9%	Intramuscular	The United States
	Manivannan et al.,^(^ ^14^ ^)^	33.0% and 51.0% (before and after intervention)	Not specified	The United States
	Manuyakorn et al.,^(^ ^19^ ^)^	93.8%	Intramuscular	Thailand
	Plumb et al.,^(^ ^20^ ^)^	45% and 74% (2002 and 2013, respectively)	Intramuscular	The United Kingdom

Three studies approached the local application of IM adrenaline. Two hospitals in England, opted for application in vastus lateralis muscle of the thigh,^(^ ^5^ ^)^ 31.1% and 43%, respectively. These hospitals site option was higher than among primary care physicians from Turkey (28.7%)^(^ ^11^ ^)^ and paramedics from the United States (11.6%).^(^ ^6^ ^)^

Other investigation by these studies from England was related with the correct adrenaline dosage (0.5mg in adults),^(^ ^5^ ^)^ in the two hospitals (37.9% and 26.8%). Both results were higher than those found in Turkey that corresponded to 16.6%.^(^ ^11^ ^)^

A single study with simultaneous analysis of medication/route/correct dosages observed that only 14.4% of physicians administered IM adrenaline of 0.5mg dosage in adults in the vastus lateralis muscle of the thigh, and this information totally agree with care guidelines of anaphylaxis.^(^ ^5^ ^)^

In ibero-american countries, 12.3% and 30.6% of specialists and non-specialists, respectively, confirmed the adrenaline administration only in patients in shock.^(^ ^9^ ^)^

### Prescription of adrenaline auto-injector

Six studies observed adoption of adrenaline auto-injector by physician. Four studies were from the United States, one from Turkey, and one from Thailand. The frequency of this strategy prescription ranged from 39.2% to 100% among physicians, and frequency was visibly higher among specialists ( Table 3 ).

**Table 3:** Frequencies of adrenaline auto-injectors prescriptions

Authors	Adrenaline auto-injectors prescription frequency	Country
Fineman et al.,^(^ ^8^ ^)^	99% allergists/immunologists	The United States
Erkoçoğlu et al.,^(^ ^11^ ^)^	39.2% non-specialized primary care physicians	Turkey
Manivannan et al.,^(^ ^14^ ^)^	54% before and 62% after non-specialist intervention	The United States
Wang et al.,^(^ ^15^ ^)^	72.7% non-specialist	The United States
Altman et al.,^(^ ^18^ ^)^	100% allergists/pediatricians, 93% allergists, internists, 88% family physicians, 63 emergency physicians	The United States
Manuyakorn et al.,^(^ ^19^ ^)^	40.2% non-specialist	Thailand

In a Turkish study,^(^ ^12^ ^)^ only 20.3% of professionals who participated in the study were aware about the existence of adrenaline auto-injectors. The study included physicians, nurses, paramedics, and medical students, but results were not organized by professional category.

### Recognizing anaphylaxis signs and symptoms

Five studies investigated recognition of anaphylaxis signs and symptoms. Again, a predominance of North American publication was seen (3), followed by Turkey (1) and the United Kingdom (1).

Interviews were carried out by phone with North American physician including allergists and immunologists, emergency physicians, family physicians and pediatricians. Respiratory problems were more the most mentioned affection (71% to 77% of the sample), followed by dizziness/faint (52 to 68%), edema (38 to 54%) and skin reactions (from 26 to 56%).^(^ ^18^ ^)^ Authors highlighted lacks of knowledge about anaphylaxis mainly evident between physician who had emergency and primary care.

However, the application of the questionnaire found that 84.7% of participants signed correctly the major signs and symptoms of anaphylaxis.^(^ ^12^ ^)^ Still, the authors who did not highlight most remembered sings. An important note is that this result reflects the opinion of group composed by physicians, nurses, paramedic, and medical students.

Paramedics from the United States who were e-mail interviewed using a questionnaire. In this study, 98.9% of participants correctly recognized a case of anaphylaxis, and only 2.9% correctly identified its atypical form.^(^ ^6^ ^)^

Hypothetical clinical cases were used to check the participants' ability to recognize anaphylaxis evidences. Of these, 84.9% of physicians recognized correctly the affection correlation with skin eruptions with pruritus and respiratory difficulty. In addition, 60.9% of professionals did the right diagnosis of anaphylaxis when they signed the option that identified hypertension in children, and dizziness after consumption of peanuts.^(^ ^15^ ^)^

The same method was used in a study from the United Kingdom that tested knowledge of physician in a sample of five clinical cases: 100% of participants identified the single case that diagnosis was anaphylaxis, and they adequate reported signs and symptoms: skin eruptions, dyslexia, wheezing, and hoarseness after consumption of seafood.^(^ ^20^ ^)^

### Patient in observation status after resolution of the anaphylaxis

Four studies raised the need of keeping patient in observation status for a period after resolution of the anaphylaxis picture: one in Turkey,^(^ ^12^ ^)^ two in the United States^(^ ^13^ ^,^ ^14^ ^)^ and one in ibero-american countries.^(^ ^9^ ^)^

The studied sample in Turkey observed that less than half of physicians (47.4%) considered place the patient in observation status for a period of at least 6 to 8 hours after stabilization.

Another study that included pediatricians from the United States showed that 40.4% of them were from a teaching hospitals, 35.7% from a hospital with medical residency program (without specify the specialty), and 26% were from another hospital but without medical residency program. All participants of this study reported to place the patient in observation status after anaphylaxis resolution.^(^ ^13^ ^)^

A retrospective study that analyzed medical records before and after implementation of a consensus about anaphylaxis in the emergency department showed that the practice to keep the patient under surveillance after resolution of anaphylactic picture increased from 44% to 65%.^(^ ^14^ ^)^

On the other hand, in a sample including ibero-american countries to place patients in observation status was more frequent. The study reported that 91.7% of allergists and immunologists placed their patient in observation status from 6 to 8 hours; and this result was higher than the one found among non-specialties (83.1%).^(^ ^9^ ^)^

### Other findings

Two studies from Turkey including pediatricians reported that percentage of participants who correctly answered about reversion of mild and severe anaphylaxis was 11.3% and 3.2%, respectively.^(^ ^16^ ^)^

The impact of promote information in clinical practice guidelines regarding anaphylaxis care in a researching hospital was checked by using pre- and post-training questionnaires and a score system. There was a significant increase in knowledge of residents in family medicine 10 weeks after intervention (score from 34.4 to 58.2; p=0.032).^(^ ^7^ ^)^

## DISCUSSION

The concern with knowledge of physicians about the use of adrenaline was highlighted in most of studies on anaphylaxis published in the last 5 years. As a consequence, this drug become consolidated and pharmacological studies, clinical observations, and also clinical trials using animal model within the last 30 years in the international scenario,^(^ ^21^ ^)^ have indicated this medication as the first choice for emergency treatment of anaphylaxis.^(^ ^22^ ^)^ The lack or delay medicine administration can cause irreversible harms to the patient.^(^ ^21^ ^)^

Based on studies that analyzed frequency of adrenaline use, mainly by IM approach, better results have been observed among allergists and immunologists from the United States. However, among non-specialists or specialists from other areas in the country the same performance is not observed. Better results on this regard are observed in Thailand and the United Kingdom in which family medicine is better structured.

A relevant observation is the difference in frequency of IM adrenaline use in the studied countries. Perhaps, this difference is due to the unequal distribution of information in international guidelines on management of anaphylaxis; this information was stated in a qualitative study.^(^ ^17^ ^)^ A multicenter study including Brazilian centers^(^ ^9^ ^)^ reported that 23.8% of non-specialists physicians favored IM adrenaline. This result indicate the little knowledge on IM adrenaline by professionals in Brazil compared with those in the United States,^(^ ^6^ ^,^ ^13^ ^,^ ^14^ ^)^ England/the United Kingdom,^(^ ^5^ ^,^ ^20^ ^)^ Turkey,^(^ ^11^ ^,^ ^12^ ^)^ and Thailand.^(^ ^19^ ^)^

Of sample with specialized and non-specialized physicians 12% to 30% of them, respectively, did affirmed to administrate IM adrenaline only in shocked patients, not when symptoms appear – when great opportunity exist to prevent the shock.

Countries with higher level of knowledge about adrenaline auto-injector and its adequate route of administration are still subjects to the little knowledge about the other aspects, for example, England,^(^ ^5^ ^)^ in which 77% of non-specialists physician mentioned IM adrenaline, only 37% of them would use it on the vastus lateralis muscle, and 32% would use a dosage of 0.5mg, an amount that can also compromise patient care.

The guidance regarding the use of adrenaline^(^ ^23^ ^)^ auto-injectors must be stimulated,^(^ ^24^ ^)^ specially for patients with idiopathic anaphylaxis or when there is continuous risk of exposition to triggers difficult to be prevented.^(^ ^3^ ^)^ Studies found showed that use of auto-injectors was higher in the United States, although few data on this regard were found in other countries.

Low frequency of adrenaline auto-injector prescription in countries that this product is available constitutes a lack of attention toward technology that already exists, but is not available in other countries. Other reason to discourage its prescription is the high cost. The cost can be a negative influence regarding frequencies in Turkey^(^ ^11^ ^)^ and Thailand,^(^ ^19^ ^)^ only countries outside the United States in which a comparison was found regarding adrenaline auto-injectors prescription.

The adrenaline auto-injector is still not commercially available in Brazil.^(^ ^9^ ^)^ In the United States, a country that this medication is broadly available,^(^ ^9^ ^)^ almost all allergists and immunologists confirmed its prescription. This information confirms the high acceptance of this new technology and its adoption in care during healthcare practice.

The unsatisfactory knowledge observed, which most of intervention studies done in the United States^(^ ^14^ ^)^ and Turkey^(^ ^7^ ^)^ proved the increase in the understanding of physicians after dissemination of information in the World Allergy Organization guidelines, which represent an action that must be urgently encourage to improve care to this clinical picture.

The knowledge of anaphylaxis evidences, the third most pointed factor by authors, consists of a crucial point for immediate care; considering that this affection is generally clinically diagnosed.

The differences in methods used by studies did not enable precisely comparisons regarding physicians' performance, especially because studies also included other professionals and students. The complexity of diagnosis should be considered when this clinical picture is discussed.^(^ ^17^ ^)^

The follow-up of a patient after an episode of anaphylaxis is essential to prevent fatal outcome because of biphasic reaction, which is the second episode of anaphylaxis.^(^ ^25^ ^)^ In most of the cases, the patient is followed-up for up to 8 hours after resolution of the initial event,^(^ ^3^ ^)^ including cases without new contact with the triggering agent. Patients should be placed in observation status in the emergency unit.^(^ ^21^ ^)^ A study reported that physician from ibero-american countries pay more attention to follow-up than those from the United States and Turkey.

A limitation of our study was to focus on the four most approached aspects (medicine and route of administration, use of auto-injectors, identification of signs and symptoms, and time of observation), given the heterogeneity of information presented by authors and methods used by them relevant other scientific results may not be included in the analysis.

## CONCLUSION

According to recent literature, adrenaline was more frequent used by allergists and immunologists than other specialists.

The United States had the highest frequency of adrenaline autoinjector prescription than other countries included in our analysis.

The complexity of recognize signs and symptoms of anaphylaxis characteristics was reflected in the assessments used by authors, and this complexity also prevented precise comparisons among studies included in our analysis.

Patient follow-up after resolution of anaphylaxis was higher in ibero-american countries than in the United States and Turkey.

We observed that knowledge about anaphylaxis diagnosis and treatment is unequal promoted in a number of countries, this knowledge is even lower in ibero-american countries. This study emphasizes the need to promote international guidelines on diagnosis and management of anaphylaxis among non-specialists as well as to provide adrenaline auto-injector in countries in which this device is not available in order to prevent fatal outcomes.
